# Community-based postnatal care services for women and newborns in Kenya: an opportunity to improve quality and access?

**DOI:** 10.7189/jogh.11.07006

**Published:** 2021-03-10

**Authors:** Brady Burnett-Zieman, Timothy Abuya, Daniel Mwanga, John Wanyugu, Charlotte E Warren, Pooja Sripad

**Affiliations:** 1 Population Council, Washington, D.C., USA; 2 Population Council, Nairobi, Kenya; 3 Ministry of Health, Division of Community Health Services, Kenya

## Abstract

**Background:**

In resource-constrained settings, Community Health Workers (CHWs) are the first point of contact between communities and the health system, as providers of maternal and newborn health services. However, little is known of the quality of community-based postnatal care (PNC). We assessed the content of PNC provided by CHWs and women’s experiences of care in two Kenyan counties.

**Methods:**

We used a cross-sectional, mixed methods design to examine the quality of PNC services provided by CHWs. Trained observers attended PNC home visits to assess technical quality using a 25-item checklist covering four PNC domains: infant health warning signs, maternal health warning signs, essential newborn care, and breastfeeding. The observers completed an 8-item communication quality checklist. We conducted follow-up surveys with observed PNC clients to assess their experiences of care. Finally, we used in-depth interviews with CHWs and focus group discussions with observed PNC clients to understand the experiential quality of care.

**Results:**

Observations suggest shortcomings in the technical quality of PNC home visits. CHWs completed an average of 6.4 (standard deviation SD = 4.1) of the 25 PNC technical quality items. CHWs often lacked essential supplies, and only six percent carried all four of the CHW job aids and tools specified in the national guidelines for maternal health at community level. However, CHWs completed an average of 7.3 (SD = 1.1) of the 8 communication quality items, and most PNC clients (88%) reported being satisfied during follow-up interviews. Higher technical quality scores were associated with older mothers, better communication, longer visit duration, and CHWs who carried at least three job tools. CHWs expressed a strong sense of responsibility for care of their clients, while clients underscored how CHWs were trusted to maintain their clients’ confidentiality and were a valuable community resource.

**Conclusion:**

This study identified gaps in the technical quality of CHW PNC practices, while also recognizing positive elements of experiential quality of care, including communication quality, and trusting relationships. This study also demonstrated the strength of the CHWs’ role in establishing linkages between the community and facilities, as long as the CHW are perceived as, and enabled to be, an integral part of the PHC network in Kenya.

Over the past decade, increases in the proportion of women who receive antenatal and childbirth care from skilled health professionals has yielded significant global reductions in maternal and newborn morbidity and mortality [1-4]. Despite these gains, many women around the world still lack access to high-quality postnatal care (PNC) services, slowing overall improvements in health outcomes for mothers and infants [5-8]. In many resource-constrained settings, trained community health workers (CHWs) are often the first point of contact between communities and the health care system. They play a vital role in conveying essential health information in a culturally sensitive manner, empowering community members to make informed decisions and improving community-level access to essential health services [9-11].

Ensuring that CHWs can provide accurate and timely health information and deliver high-quality counselling services to women during the immediate postnatal period is necessary and has the potential to reduce maternal and infant illness and death [12]. However, the quality of care that CHWs provide – particularly during the postnatal period – is often poorly measured. There is need to better understand the nature and quality of care that CHWs provide and how they might be better supported [13]. Quality of care encompasses provision of technical quality and experience of care [14], both of which can also be used to understand quality of community-based care.

Assessing the quality of interactions between CHWs and community members is important in understanding how the services that CHWs provide affect the health outcomes of community members. Client satisfaction with services is often used as a proxy measure for quality of care provided by CHWs; however, as programs scale up, measuring the technical quality of the services delivered becomes critical [15]. Prior studies have identified challenges in assessing the impact of CHW programs on service quality, due, in part, to design limitations [16,17] and lack of standardized quality assessment metrics [18]. This paper reports on quality of care measured by process and experience of maternal and young infants (under 60 days old) care services provided by CHWs in Kenya.

## Community health services in Kenya

Although Kenya has increased efforts towards achieving the Sustainable Development Goals (SDG) targets of less than 70 maternal deaths per 100 000 live-births and 12 neonatal deaths per 1000 live births by 2030 [19,20], the number of deaths remain relatively high [21]. One strategy being championed to reduce maternal and neonatal mortality is to increase access to services at lower levels of the health system, especially at the community level [22]. Kenya has a four-tier health system structure with the vision of strengthening and re-emphasizing the focus on a primary health care (PHC) network. Community health services (CHS) are articulated as first-tier health services in the Ministry of Health (MoH) policies [22-24], and are embedded within the PHC concept [25]. The first tier is represented by Community health units (CHUs), each of which serves a catchment population of 5000 people and is staffed by 10 volunteer CHWs (referred to in Kenya as community health volunteers – CHVs) and supervised by a community health extension worker (CHEW). CHEWs are qualified individuals that are employed by the formal health system to provide training and technical support to CHVs. Each CHEW supports approximately 25 CHVs. The CHVs are chosen by their community to provide home-based care and counselling services. Through their activities, they create demand for formal clinical services and link clients to second-tier PHC services at dispensaries and health centers [25]. As CHVs support early identification and treatment of diseases and are directly aware of the needs of their community, they are often perceived as role models and recognized as champions for health-related matters. Evidence suggests that CHS in Kenya increases attendance of at least four antenatal care visits, deliveries by skilled birth attendants, intermittent preventive treatment, testing for HIV during pregnancy, exclusive breastfeeding, and hygiene practices [26,27].

In this context, we examined the technical and experiential quality of PNC for women and their babies provided by CHVs in Kenya using observations of home visits and follow up surveys. We describe data on the scope of PNC care provided by CHWs to women and infants and document underlying drivers of quality care. We compare the expected practices using national guidelines for providing maternal and infant health services at community level [28].

## METHODS

The research presented here is part of the Frontline Health project, which focuses on ways to measure CHW per­formance and identify the operational considerations that affect the institutionalization of community health in an array of settings. Using a cross-sectional, mixed methods design, this study examines the quality of PNC services provided by CHVs to women and young infants (under 60 days) in two counties in Kenya. The research team captured data from three sources: direct observation of PNC home visits to assess technical quality, follow-up surveys with observed PNC clients to assess experiential quality, and a series of in-depth interviews (IDIs) with CHVs and focus group discussions (FGDs) with observed PNC clients to develop a comprehensive understanding of the technical and experiential qual­ity of the care, including contextual factors that influence quality of care. This study uses three data sources: direct observation of PNC home visits to assess technical quality, follow-up surveys with observed PNC clients to assess experiential quality, and a series of in-depth interviews (IDIs) with CHVs and focus group discussions (FGDs) with observed PNC clients to develop a comprehensive understanding of the technical and experiential quality of the care, including contextual factors that influence quality of care.

### Study sites and sample

The study was conducted in rural settings in Kilifi (coastal) and Bungoma (western) Counties. Both counties reflect a range of commonly observed barriers to care, including geographical access constraints and cultural vulnerabilities. Kilifi has a neonatal mortality rate of 26 deaths per 1000 live births, a skilled delivery rate of 52.3%, an under-five mortality rate of 141 deaths per 1000 live births, and a maternal mortality ratio of 540 deaths per 100 000 live births. Bungoma has a neonatal mortality rate of 33 deaths per 1000 live births, a skilled delivery rate of 44%, an under-five mortality rate of 145 deaths per 1000 live births, and a maternal mortality ratio of 360 deaths per 100 000 live births [29]. Kilifi and Bungoma Counties were purposefully selected in collaboration with County Health Management Teams to include a functional CHS where CHVs conduct routine visits, collect data, and receive some form of incentives. Additionally, the selected sites had several collaborative projects between non-governmental organizations (NGOs) and the government being implemented to strengthen local CHS through capacity building and improve access to health information.

### PNC home visit observations

From 29 July to 23 August 2019, we shadowed 68 CHVs – 32 in Kilifi County and 38 in Bungoma County – as they conducted PNC home visits with postnatal women whose infants were less than 60 days old. CHVs were linked to 12 facilities representing different tiers of the health system (eg, referral hospital/one sub-county hospitals, health centers, dispensaries); three quarters were female. We observed between 1-3 PNC home visits with each CHV. During observations, trained research assistants with social science backgrounds completed an observation guide without intervening during the home visit.

Observers measured technical quality of care using a checklist of 25 key health items that were adapted from the national CHV Manual [28] and are routinely completed by CHVs during a PNC visit. These items comprise four domains: infant health warning signs, maternal health warning signs, essential newborn care, and breastfeeding. Observers directly assessed whether CHVs completed these 25 items during PNC home visits. The observations also assessed CHV’s communication with their clients, whether follow-up actions were taken when an infant was identified as unwell, and whether CHVs were properly equipped with four essential job tools: referral slips for follow-up care, a visit schedule register, the CHV household handbook, and a set of community health job aids.

### PNC client follow-up survey

Within two weeks of completing each observation, we conducted follow-up surveys with the observed PNC clients. During the follow-up interviews, we collected basic demographic information and assessed the client’s experience of the observed CHV visit, including the four PNC domains and their communication with the CHV. Interviewers asked women about their perceptions of the 25 health checks assessed during the observations and asked a series of communication quality items about the social interaction and attitudinal aspects of care. Interviewers also asked women about their satisfaction with the care they received from the CHV using a Likert-scaled item with four ordinal responses ranging from very dissatisfied to very satisfied.

### Qualitative interviews

To better understand the contextual factors that influence CHVs ability to plan and conduct PNC visits, we held IDIs with 12 of the CHVs who were observed during a PNC visit and eight of the CHEWs who supervise them. We also conducted two FGDs in each county (n_total_ = 4), with a total of 29 postnatal women whose CHV home visits were observed for this study. Trained qualitative interviewers facilitated IDIs and FGDs using pre-developed interview guides.

### Analysis

We present cross-sectional data from the technical and communication quality checklists as observed during PNC home visits. Specifically, we examined the proportion of visits at which each item in the checklists were completed and produced summary scores for each domain. As some checklist domain scores might improve over time, while others – particularly those in the neonatal care and breastfeeding domains – would logically be expected to decrease as the child ages, we disaggregated the observation data by the number of contacts the mother had received from a CHW at the time of the observation: first contact, second contact, and third or greater contact. We used simple linear regression to assess whether there is any relationship between aggregated quality scores and contact number. Finally, we conducted multivariate linear regression analyses to examine factors associated with observed quality of care, controlling for county, CHV sex, maternal age, whether the infant was unwell during the visit, visit duration, and whether the CHV had carried at least three job tools to the visit. As CHVs conducted multiple PNC home visit observations, we used cluster analysis methods to adjust for intra-class correlation. All data were analyzed using Stata 14 (Stata Corp, College Station, TX, USA).

For qualitative data, we thematically analyzed the transcripts, field notes, and observations using NVivo 12 (QSR International, Victoria, Australia) software. We developed and refined an initial coding framework based on the process of open coding and progressive categorization of issues. We used charting processes to summarize key themes and concepts including references and quotations that we compared across sites for similarities and differences to support identification of key issues around quality of care for PNC clients.

## RESULTS

### Sample demographics

We observed a total of 166 PNC home visits (Kilifi: n = 82; Bungoma: n = 84) that lasted an average of 23 minutes, with an average of 2.2 PNC visits per CHV (standard deviation (SD) = 0.73; range = 1-3 visits). Follow-up interviews were completed with 148 (Kilifi: n = 70; Bungoma: n = 78) of the observed clients. According to the MoH guidelines, postnatal women and their infants are supposed to receive visits at least seven times after birth, with the first visit during week one (day one, day three, or day seven, regardless of place of delivery), the second contact at one month, the third contact at six months, the fourth contact at nine months, the fifth contact at 12 months, the sixth contact at 18 months, and the seventh contact at 24 months [28].

Of the observed consultations (**Table 1**), 31% were the first contact between the woman and CHV after giving birth, 31% were second contacts, and 36% were the third or greater contact between the CHV and woman and her infant under 60 days. Median infant age was 28 days (IQR = 14-43 days) and ranged from 0 to 61 days. Median infant age increased from the first contact (22 days, IQR = 10-37 days) through to three or more contacts (34 days; IQR = 21-43 days). Maternal age ranged from 16 to 43 years; median age was 26 years (IQR = 22-31 years).

**Table 1:** Demographic characteristics of CHVs and women (clients) observed

Indicator	N = 148	%
**CHV gender:**		
Female	116	78.4%
Male	32	21.6%
CHV facility association:*		
Hospital	43	29.1%
Health Centre	18	12.2%
Dispensary	87	58.8%
**Mother's age, years (median, IQR)**	26	(22-31)
Infant's age, days (median, IQR) *	28	(14-43)
**Mother's highest level of schooling**		
Never attended	11	7.4%
Primary	79	53.4%
Secondary	45	30.4%
Tertiary	13	8.8%
**Mother's religion:**		
Christian	126	85.1%
Muslim	21	14.2%
Traditional	1	0.7%
**Mother's marital status:**		
Never married	15	10.1%
Married/cohabitating	128	86.5%
Divorced/separated/widowed	5	3.4%
**Location of childbirth:**		
Health facility	133	89.9%
At home	8	5.4%
At home with TBA	3	2.0%
In-transit to health facility	4	2.7%

CHV – community health volunteer, IQR – interquartile range, TBA – traditional birth attendant

### Provision and experience of PNC

During observed home visits, CHWs completed an average of 6.4 (25.6%) of the 25 PNC technical quality checklist items (**Table 2**). The scores did not vary significantly by contact number or CHV gender (data not shown). CHVs performed best on items within the breastfeeding domain, completing a per-visit average of 3.7 (61.7%) out of six possible items. Low overall observed care scores suggest that CHVs completed very few of the items in any of the three remaining domains. On average, CHVs checked 1.3 (14.4%) of the nine infant danger signs and provided information on 0.8 (16%) of essential newborn care items. Of the items specified in the checklist, infant temperature was checked most frequently (32.4% of visits), followed by feeding (23.0% of visits). CHVs rarely checked any postnatal danger signs for the mother’s health, averaging 0.5 (10.0%) of five possible items. When examined across contact numbers, we find a small but significant increase (0.581 per visit, *P* = 0.01) in the total number of infant health items completed between the first contact through the third or more. However, other domains and the total technical quality of care score do not appear to exhibit relationships with contact number. This is further evidenced when individual checklist items are examined by contact number. The proportion of visits at which a given item is addressed often varies considerably between contacts, suggesting little systematic relationship with the timing of completing each item. For instance, many of the items in the neonatal care domain, which are most relevant immediately after birth, were assessed more frequently during the third or greater contact than at the first.

**Table 2:** Postnatal care (PNC) domains and indicators by contact

PNC domain indicators	First contact		Second contact			Third + contact				Total				Trend analysis	
**N = 47**	**%**	**N = 47**	**%**	**N = 54**			**%**	**N = 148**		**%**		**Coef. (se)**		***P*-value**
a. Temperature based on mother's report of touch (feel cold or hot)	11	23.4	17	36.2	20			37.0	48		32.4				
b. Only moves when stimulated, or does not move even on stimulation	1	2.1	7	14.9	6			11.1	14		9.5				
c. Bleeding from the umbilical stump	4	8.5	9	19.1	10			18.5	23		15.5				
d. Yellow sole/eyes	4	8.5	8	17.0	5			9.3	17		11.5				
e. Low weight gain	5	10.6	8	17.0	13			24.1	26		17.6				
f. Fast breathing (defined as two counts of 60 breaths)	4	8.5	10	21.3	8			14.8	22		14.9				
g. Convulsed or fitted since birth	3	6.4	6	12.8	6			11.1	15		10.1				
h. Severe chest in-drawing (defined as chest draws in as the baby breathes)	0	0.0	1	2.1	4			7.4	5		3.4				
i. Not able to feed since birth, or stopped feeding well	10	21.3	10	21.3	14			25.9	34		23.0				
**Total observed infant health score (mean, 95% CI)**	**0.9**	**0.4-1.4**	**1.6**	**1.1-2.2**	**1.6**			**1.1-2.1**	**1.3**		**1.1-1.7**		**0.581 (0.22)**		**0.010**
a. Presence of high fever	4	8.5	7	14.9	4			7.4	15		10.1				
b. Heavy vaginal bleeding	5	10.6	7	14.9	5			9.3	17		11.5				
c. Lower abdominal pain and foul-smelling discharge	5	10.6	6	12.8	10			18.5	21		14.2				
d. Severe headache, blurred vision	5	10.6	5	10.6	2			3.7	12		8.1				
e. Convulsions or fits	2	4.3	3	6.4	2			3.7	7		4.7				
**Total observed maternal danger signs score (mean, 95% CI)**	**0.4**	**0.1-0.8**	**0.6**	**0.3-0.9**	**0.4**			**0.1-0.7**	**0.5**		**0.3-0.7**		**0.086 (0.14)**		**0.534**
a. Drying the baby as soon as the baby is born	2	4.3	5	10.6	3			5.6	10		6.8				
b. Keeping the room warm where the baby is to be born	8	17.0	13	27.7	17			31.5	38		25.7				
c. Keeping the baby in skin to skin contact with the mother	8	17.0	8	17.0	15			27.8	31		20.9				
d. Putting the baby to the breast as soon as the cord is cut	8	17.0	11	23.4	12			22.2	31		20.9				
e. Not bathing the baby on the day of birth, but cleaning with a cloth	2	4.3	6	12.8	7			13.2	15		10.2				
**Total observed essential newborn care items checked (mean, 95% CI)**	**0.6**	**0.2-1.0**	**0.9**	**0.6-1.3**	**1**			**0.7-1.3**	**0.8**		**0.6-1.0**		**0.241 (0.14)**		**0.096**
a. The baby should not be fed on anything apart from breast milk in the first six months	47	100.0	44	93.6	50		92.6			141		95.3			
b. Giving other food or fluids can make the baby very sick	35	74.5	34	72.3	34		63.0			103		69.6			
c. The first yellow milk (colostrum) acts as the babies first immunization	19	40.4	15	31.9	18		33.3			52		35.1			
d. Immediate suckling to help mother make more milk	18	38.3	19	40.4	22		40.7			59		39.9			
e. Hand washing before touching or breastfeeding the baby, eating or visiting	31	66.0	27	57.4	35		64.8			93		62.8			
f. Clean home environment	30	63.8	28	59.6	35		64.8			93		62.8			
**Total observed breastfeeding score (mean, 95% CI)**	**3.8**	**3.3-4.3**	**3.6**	**3.0-4.1**		**3.5**	**3.1-4.1**			**3.7**		**3.4-3.9**		**-0.092 (0.20)**	**0.654**
**Total observed quality of care (mean, 95% CI)**	**5.8**	**4.6-6.9**	**6.7**	**5.5-7.8**		**6.6**	**5.5-7.7**			**6.4**		**5.7-7.0**		**0.816**	**0.089**

CI – confidence interval

CHVs did not confirm whether the child was unwell at one-third of observed visits (*data not shown*, 37.8%). Among the 92 visits where the CHV did confirm the child’s wellness, 11 (12%) subsequently checked the child for additional symptoms, and seven referred a child for additional health services; the most common symptoms were inability to pass urine or stool (n = 5) and restlessness (n = 3).

Qualitative data from women who received PNC from CHVs reinforce the emphasis on breastfeeding as a major area of focus (**Table 3**); not only do CHVs explain in detail how to continue this practice, but they are able to situate it within the broader context of a woman’s own nutritional status and life circumstances. Despite the low technical quality scores on the maternal and newborn danger signs and essential newborn care domains, quali­tative data suggest that some CHVs do check on other health issues that may require referrals. Examples of referrals for pneumonia, immunization, malaria, or other illnesses emerged (**Table 3**). Interviews with CHVs described a broader set of duties than are adequately captured by the PNC checklists.

**Table 3:** Qualitative findings across PNC domains

Domain	Sample quotation
Infant danger signs	*“After giving birth at home, the CHV comes to check if you are taking good care of the baby, keeping him warm or is pneumonic and refer you to the hospital…R4: There are some when they give birth at home and not take the child for immunization so the CHV will advise her to take the child to the clinic to be immunized.”* (FGD, PNC women, Bungoma)
Maternal danger signs	*“They [CHVs] have test kits for malaria diagnosis, if you are feeling unwell, they test you and give medicines….they also have these drugs…or if they don’t, they test you and write a referral to come to the hospital to consult a health worker*.”(FGD, PNC women, Bungoma)
Essential newborn care	*“She [CHV] tells us that the baby should be fed nothing else except breast milk, so I followed it and I have seen the outcome because my baby does not easily get sick and grew well, so I saw that to be true.”* (FGD, PNC women, Kilifi)
	*“If she [mother] is young he [CHV] can give more advice because she doesn’t know more about taking care of baby, like hygiene… but for the older person she has experience hence he may be harsh.”* (FGD, PNC women, Bungoma)
Breastfeeding	*“She [CHV] taught about nutrition while breastfeeding. She said we [mothers] should eat five times in a day so that the baby can get enough milk. And every time you eat a balanced diet and a fruit must always be a part of every meal you are eating, and she said that for you to be eating a balanced diet, you don’t have to be buying everything from the shops, you can just look for the food that is available in the gardens and eat those so that the baby can get enough food.”* (FGD, PNC women, Kilifi)

FGD – focus group discussion, PNC – postnatal care, CHV – community health volunteer

“We usually have topics that we talk to them about that we have not been told by the facility, for example, malaria when it is the rainy season, or nutrition. You go to the community to those households; you sit down with the members and you discuss. During these sessions if a problem arises, you might look at the baby’s clinic book and find that she [mother] has defaulted and has not taken the baby to the clinic.” *– IDI, CHV*

### Communication quality in observed and experienced in PNC

Moreover, while CHVs performed poorly on technical quality, they performed better on basic communication skills. Quantitatively, CHVs scored an average of 7.3 on the eight-item communication quality checklist during observed PNC (**Table 4**). Items most frequently missed were asking the woman whether she had any questions (16.2%) and using simple words in the local language (20.3%).

**Table 4:** Communication quality and CHV capacity

Communication quality and CHV tool indicators	First contact		Second contact				3rd + contact			Total		Trend analysis	
	**N = 47**	**%**		**N = 47**	**%**	**N = 54**		**%**	**N = 148**		**%**	**Coef/OR (se)**	***P*-value**
Greet the client	47	100.0		47	100.0	54		100.0	148		100.0		
Explain purpose of visit	44	93.6		43	91.5	52		96.3	139		93.9		
Respectful throughout the interaction	47	100.0		47	100.0	54		100.0	148		100.0		
Spoke gently to the family	47	100.0		46	97.9	51		94.4	144		97.3		
Ask the woman if she has any questions	38	80.9		38	80.9	48		88.9	124		83.8		
Use simple words in local language	35	74.5		37	78.7	46		85.2	118		79.7		
Answer questions simply	44	93.6		39	83.0	51		94.4	134		90.5		
Thank the family for the visit and say when CHV will return	39	83.0		43	91.5	48		88.9	130		87.8		
**Total CHV communications score (mean, 95% CI)**	**7.3**	**6.9-7.6**		**7.2**	**6.9-7.6**	**7.5**		**7.0-8.0**	**7.3**		**7.2-7.5**	**0.194 (0.11)**	**0.088**
Had all tools	1	2.1		3	6.4	5		9.3	9		6.1	2.440 (0.90)	**0.019**
Had any tools	26	55.3		36	76.6	36		66.6	98		66.2		**0.46**
-Referral slips	20	42.6		25	53.2	29		53.7	74		50.0		
-Visit schedule register	17	36.2		29	61.7	30		55.6	76		51.4		
-Household handbook	7	14.9		10	21.3	10		18.5	27		18.2		
-CHV job aids	9	19.1		15	31.9	15		27.8	39		26.4		

CI – confidence interval, OR – odds ratio, CHV – community health volunteer

Qualitatively, most CHVs felt that they were able to communicate freely and effectively with postnatal women. CHVs described developing a trusting CHV-client relationship where a client feels secure as critically important in empowering women to be active participants in their care. They concurred that first-time mothers are counselled differently than others, with greater emphasis on danger signs. The CHVs also mentioned the importance of health facility nurses reinforcing the material that is initially presented by the CHVs during routine home visits.

“When a [CHV] explains to them and gives good counsel, they understand and follow whatever you tell them… [also] there are no challenges when [some]one gets pregnant… they remember when someone [else] was [pregnant], and [the CHV] told me this and that.” *– IDI, CHV*

“At the community, it happens during the household visits… That is when we sit down with the mother and talk to her; if she is pregnant you talk to her about the importance of going to the clinic, the importance of delivering at a health facility and eating a balanced diet. If she is a mother of a young child, you also show her how to breastfeed the baby well, to use family planning and all that. So, it can either be in a group or it can be done one on one.” *– IDI, CHEW*

Additionally, CHVs described feeling responsible for ensuring that their clients were provided with necessary facility-based services by personally bringing them to the facility or supporting their clients at personal expense.

“If you see that she is completely unable to afford anything, as a CHV, it is your responsibility to also help them by either bringing them to the hospital or providing for them money for the hospital and the items required if you can afford to.” *– IDI, CHV*

Experiential quality of interactions as described by follow-up survey findings (data not shown) suggest that most clients (87.8%) were satisfied with the care they received from the CHV. Only 12.2% reported being dissatisfied (7.4%; n = 11) or very dissatisfied (4.7%; n = 7). Satisfaction appeared to improve as contacts increased. Nearly one quarter of participants who were observed during a first contact reported dissatisfaction, while none of the participants who were observed during later contacts (three or more) reported being dissatisfied with the care received from the CHV. This trend, however, was only marginally significant (*P* = 0.052). Among the 18 women who reported being dissatisfied, seven cited the CHV taking insufficient time with her as the main reason they were not satisfied, and five said it was because the CHV repeated themselves or did not teach them anything new. One respondent said she was dissatisfied due to the expectation of being hospitable to the CHV while she was also caring for her newborn.

Qualitatively, clients describing factors contributing to their satisfaction or dissatisfaction with CHV care focused less on prescribed PNC wellness checks (technical quality) and felt that communication quality and trusting interactions with CHVs were more important. In the PNC context, trust manifests uniquely in relation to the reliable information and support a CHV affords both mother and infant over the course of multiple visits. Clients associate the affirming, positive and trusting relationships with CHVs in their ability to give advice in an approachable way, explain and translate doctors’ and nurses’ messages, refer and accompany clients to facility-based care, and follow up to ensure positive sustained outcomes (**Table 5**). Several clients drew on the notion of confidentiality to report feeling they could speak freely with their CHV and would trust them with handling personal issues discretely and effectively (handling sensitive topics like postnatal family planning), while others were more reluctant to share sensitive information with CHVs who they perceived as untrustworthy. Clients also described some factors, such as negative quality of care or poor health outcomes at a referral facility (untimely or incomplete response or infant death), that were beyond the control of the individual CHV, but nevertheless contributed to sense of ‘broken’ trust (**Table 5**).

**Table 5:** Women’s perspective of communication quality and trusting interactions in PNC

Theme	Sample quotations
Friendly nature and approachability	*“R4: It is because she visits us and advises us…R6: she normally visits us and teaches us about how to bring up our child very well*.” (FGD, PNC women, Kilifi)
	“*R7: You will know by the tone of her voice, or say her reaction to you, how you take like when she has come with happiness… R5: She offers the services wholeheartedly.”* (FGD, PNC women, Bungoma)
Continuity of services and accompanied referrals	*“She [CHV] is near me…so if she sees something that is not going right as it should then she will come and say things like “a place where there are children shouldn’t be dirty…because of diseases”… which I find is of help to me since the hospital and the doctors are far.*” (FGD, PNC women, Kilifi)
	*“I believe because sometimes when my children are sick, he takes care of the children gives them medicine and referrals them to the clinic and I did not pay for the services.”* (FGD, PNC women, Bungoma)
	*“…[The CHV] does just refer you to go to hospital, she will come and maybe find the baby is unwell so she will ask you why is the baby unwell and yet you haven’t taken her/him to hospital. So maybe you tell her that you have not found time to go to the facility, she will take the baby and ask you to accompany her to the hospital. You will go with her until you reach the hospital and hand you to the doctor, make sure you are treated to completion and the take you back home. She will come and check on the sick baby until she makes sure she/ he is completely healed.” (*FGD, Client)
Confidentiality around sensitive topics	*“Maybe you shared confidential information with the CHV and yourself you are afraid to tell the [facility-based] service provider, and the CHV tells him/her it will be different…”* (FGD, PNC women, Bungoma)
	*“I might not be considering family planning and I get pregnant before my child reaches an age. After I should stop breastfeeding. She [CHV] advises us and let us make our own decision.”* (FGD, PNC women, Kilifi)
Negative experience or broken confidentiality	*“You can decide to go, for example the ones who are referred, they went and were attended to very fast and came back home, maybe there is someone who was referred but she still stayed for long at the hospital and maybe did not even got attended and came back home, so the following day when she meets the CHV and she [CHV] tells her [Client] to go back to the hospital, she will tell her I went like you told me and I was not attended to so I will not go. It is not like the CHV lied to her, but the problem is with where she went.*” (FGD, PNC women, Kilifi)
	*“It depends on the CHV. There are others who walk around spreading rumors while others will assure you that whatever you tell them is between you, them and the doctor they refer you to. We know them because we live with them in the village. There are others who may come to attend to you but instead deviate and start talking about others so you just know such a person you cannot tell them deeper secrets.”* (FGD, Client)

FGD – focus group discussion, PNC –postnatal care

### Confounders and context

At the start of a PNC home visit, study observers first assessed whether the CHV was equipped with four essential job tools: referral slips for follow-up care, a visit schedule register, the CHV household handbook, and a set of job aids. In only 6.1% of visits did the CHV carry all four tools. The proportion who either carried all four tools, or none did not vary significantly by visit number. Referral slips and the CHVs’ visit schedule registers were available at half (50.0% and 51.4%, respectively) of observed visits. CHVs carried job aids to 26.4% of visits and had their handbook available at 18.2% of visits.

Qualitatively, CHVs described using their schedule register to track their household visits, to make monthly PNC home visit plans, and identify the topics they will discuss during upcoming visits.

“We have referral booklets, and every CHV has 20 households… actually right now we are having 50, initially they were 20 but right now we have 50 households per CHV… or more. We usually have our work plans for the month… for instance I have them numbered, from number one up to 50; so if this month I planned to visit ten households, I will have to plan on what time I will be doing the visits and what topics will I be talking about.” *– IDI, CHV*

Other CHVs described a need for continuous training on how to use some of the technical postnatal supplies, which often depends on NGO-supported programming to supplement CHU efforts.

“Weighing scale, thermometer, and strappings to help in first aid in case someone is injured… [they train us] …At the dispensary when we have monthly meeting, they help us to know how to use them. However, I worked in hospital for some time…Mostly it is the NGOs that assist in the trainings but when the project ends, it becomes difficult. For CHEW, he cannot train us; he only calls for the monthly meeting or community dialogue meetings.” *– IDI, CHV*

In other instances, some CHVs described having enough supplies needed to do their job but suggested that the workload may be too heavy, and they would benefit from more consistent training and supervisory support. Others described visiting only a fraction of the households for which they are responsible based on the needs of each month.

To further understand factors associated with quality of care, we ran multivariate logistic regressions (**Table 6**). We found that higher quality of care scores was associated with older mothers, longer visit duration, and CHVs who carried at least three of their job tools. Significantly lower total quality scores were associated with visits where an infant was identified as unwell. A sub-analysis of individual job tools further showed that CHVs who carried the handbook typically had total quality of care scores that were about three points (Coef. = 3.13; 95% confidence interval (CI) = 0.22-6.04) higher than those who did not have their handbook. The same was true for CHVs who carried their CHV job aids (Coef. = 3.17; 95%CI = 1.05-5.28), while carrying neither the referral slips nor visit schedule registry were associated with lower total care scores.

**Table 6:** Multivariate logistic regressions exploring confounding factors on quality of CHV-provided PNC

	Total care score		
**Covariates**	**Coef.**	**95% CI**	***P*-value**
Bungoma County	-0.826	-2.30, 0.65	0.269
Male CHV	-0.535	-2.01, 0.94	0.471
Maternal age (years)	0.085	-0.01, 0.18	0.091
Infant was unwell	-1.68	-3.38, 0.026	0.053
Visit duration (minutes)	0.089	0.04, 0.14	0.001
Carried 3+ essential job tools	4.012	1.48, 6.54	0.002
	
**Covariates**	**Coef.**	**95% CI**	***P*-value**
Bungoma County	-1.74	-3.12, -0.35	0.015
Visit duration (minutes)	0.062	0.004, 0.12	0.034
Referral slips	-0.260	-1.72, 1.20	0.723
CHV register	0.884	-0.52, 2.28	0.212
Household handbook	3.125	0.22, 6.04	0.036
CHV job aids	3.165	1.05, 5.28	0.004

CHV – community health volunteer, CI – confidence interval, PNC – postnatal care

## DISCUSSION

CHVs are essential community level providers who can deliver accurate and timely health information and counselling services to women during the immediate and extended postnatal period if they are equipped adequately. This study demonstrates gaps in the technical quality of CHV practices (completion of health checks, targeting the content of the contact to postnatal women and infant’s age, use of tools, and enabling supplies). However, these gaps illustrate the importance of considering and building upon the non-technical or experiential quality elements related to strong CHV-client relationships.

Technical quality gaps reveal that CHVs completed only one quarter of the 25 prescribed PNC health checks, averaging 6.4 (95% CI: 5.7-7.1) during a home visit. These figures are consistent with similar PNC visits performed by health extension workers in northern Ethiopia [30]. However, when we examine checklist items individually, we find that the completion of any one item at a given point in time rarely follows a systematic pattern. To this end, the observation data suggests that there are no significant relationships between summary scores for the other domains – or for the total score for the checklist – and visit number. CHVs are expected to make home visits in the intermediate and extended postnatal period (as per national guidelines) [28]. Many of the 25 items included in the checklist were applicable at specific time points in the postnatal period and some only during the period immediately (48-76 hours) following childbirth (eg, essential newborn care domain, breastfeeding items including suckling and colostrum, some maternal danger signs). However, our observations indicate that many health checks, when they were completed, were performed with little consideration given to timing or appropriateness of messaging. CHVs were still discussing essential newborn care with a fifth of women whose infants were over 28 days old.

While qualitative data captured how CHVs educated first time mothers on infant danger signs, observations suggest that CHVs may be less systematic in targeting the information they present to their clients. Despite their lack of targeted counselling, CHV performance on the breastfeeding domain appears to resonate with the broader literature on their effectiveness in promoting mother-performed strategies such as skin to skin care and exclusive breastfeeding [17].

Three reasons may explain these observations. The first reason is that CHVs residential proximity and familiarity with the infants and women may lead them to prioritize components that they consider important (eg, breastfeeding) over others (eg, checking for danger signs among either mothers or infants). It is possible that CHVs intentionally skipped these items upon seeing that both mother and child were healthy-looking or covered these items at previous contacts. Second, as reflected in the qualitative data and global evidence of CHW programs, CHVs cover large catchment areas and experience increasing responsibility, making it hard to spend more time with the mothers [31,32]. These challenges, coupled with poor support for planning and efficiency, contribute to job stress and high attrition rates in CHW programs globally [31,32]. Finally, the current CHS structural norms in Kenya regard CHVs primarily as health service demand generators – namely community mobilizers that increase awareness and facilitate people’s access to medical facilities [33], rather than health workers that require supportive supervision and continuous updates of their skills. CHVs felt responsible for generating demand and ensuring that their clients completed referrals–an approach reinforced when CHVs supported their clients’ transit at personal expense. These findings are consistent with research across country settings advocating for role clarity of and increased support of CHWs as linked to improving equity, quality of care, and patient safety [34,35].

Aggregate technical quality scores appear to be influenced by the availability of job aids, illustrating the need to ensure that CHVs are equipped with essential supplies tools when conducting home visits. While technical quality gaps in CHVs’ performance of PNC checks are likely attributable to a range of factors, our data suggest that failure to bring essential supplies to PNC home visits (**Table 4**) was a common occurrence and is likely linked to low technical quality scores. CHVs most frequently carried their referral slips and visit schedule registry, enabling management of their workflow and referrals, however they carried the Household Handbook and CHV job aids less frequently. These items directly support the technical quality of CHV-led activities, including health check prompts. We found CHVs who carried three or more tools completed significantly more prescribed health checks than CHVs who lacked these items; stratified analyses by type of tool carried demonstrated that the Household Handbook and CHV job aids were principally responsible for these increases in observed technical quality of care. However, when these job aids were present, CHVs completed only a fraction of the items included in the checklist, limiting their ability to effectively target age-specific messages. Perhaps strategies to optimize the job aids in digital format may facilitate easy carry and use that can help targeted messaging.

CHVs were observed providing information on essential newborn care and recognition of infant danger signs regardless of the age of the infant. Digitization of job aids, use of mobile phones for PNC contact follow up, and further exploration of non-use may help facilitate improvements to and use of job aids. Studies suggest that simple tools can enable CHVs to plan and prioritize care, identify disparities in service delivery, and promote positive maternal and infant health outcomes [36,37]. These studies demand investment in multi-day training and continuous support for CHVs to use the job-aids effectively.

Our findings also showed that CHVs’ non-technical responsibilities often extended beyond the essential wellness checks, and CHVs perform well in the quality of communication between themselves and clients. Observations show that CHVs scored an average of 7.3 (92%) out of eight possible communication quality points during observed PNC home visits. Qualitative data validated this observation, as both CHVs and clients noted that experiential quality of interactions was considered positive, with minimal dissatisfaction of services. Where dissatisfaction was reported, it was most linked to clients who felt their CHV spent insufficient time with them, or to clients who felt their CHV repeated basic information or did not teach anything new during their visits.

CHV’s clients respected and trusted to maintain their confidentiality accrued social capital for providing safe and reliable entry to formal health services in sensitive or personally challenging situations. Future studies should examine whether facilitated referrals improve referral completion, or how trust in CHVs’ discretion may empower women to seek care for sensitive health issues. Future efforts to build capacity of CHVs and improve technical quality should not discount the less structured responsibilities carried out by CHVs and should leverage the positive community-CHV relationships[12] that characterize experiential quality of care.

Moreover, the postnatal period has long been neglected, including in health facilities in Kenya [38] where safe motherhood vouchers were available for poorer women to access postnatal services [39]. Despite some improvements in uptake of PNC from 10% in 2003 to 51% in 2014[29], many women who seek maternity services are not checked by any facility provider after childbirth [40]. It is therefore not surprising that postnatal women and their babies do not receive optimal care at the community level. Immediately, community health program planners should ensure job aids are always available and consider ways to tailor them to ensure that the timing of essential health checks and counseling messaging is consistent with the life stage of the infant. Furthermore, revising CHV PNC scope of work and increasing systemic supports to CHVs would improve technical quality of PNC and integrate it into the PHC network in Kenya [22].

### Limitations

This paper has several limitations. While our study shows that measuring technical and experiential quality of care provided by CHVs is feasible, comparative work is needed to assess transferability of the PNC observational checklist communication quality indicators (items and summary scores). The sample size of postnatal observations and client surveys was limited, and we recommend further testing in higher-powered studies in additional settings in Kenya and globally. As this study was only conducted in two counties in Kenya that have ongoing programs designed to support CHV-led activities, our results may over-estimate technical quality and are unlikely to be nationally generalizable. Similarly, although we tried to observe all CHVs attached to the health centers and dispensaries targeted by this study, there may be some level of self-selection bias wherein higher-performing CHVs would be more likely to agree to be observed. Again, such biases would likely lead to overestimates of technical quality during PNC visits. Given the cross-sectional nature of the study, the regression analysis did not consider the time-lag between which CHVs were trained on the technical module on maternal health by the MoH or the intensity of supportive supervision. This was beyond the scope of the study and would be helpful consider in future longitudinal designs.

## CONCLUSIONS

This study identified gaps in the technical quality of CHV PNC practices, while also recognizing positive elements of experiential quality of care, including communication quality, and trusting relationships. This study also demonstrated the strength of the CHVs’ role in establishing linkages between the community and facilities, as long as the CHV is perceived as, and enabled to be, an integral part of the PHC network in Kenya.
